# Supplementary material for: Colombian Essential Oil of Ruta graveolens against Nosocomial Antifungal Resistant Candida Strains
Source: J Fungi (Basel). 2021 May 14;7(5):383. doi: 10.3390/jof7050383 (PMC8156898; doi:10.3390/jof7050383)
Supplement: Supplementary file 1 [file jof-07-00383-s001.zip › jof-1144553-supplementary.pdf]

Supplementary Materials

Article

Colombian essential oil of *Ruta graveolens* against *Candida* sp. isolated from the oral cavity of patients with head and neck cancer

Matthew Gavino Donadu[1,4‡], Yeimmy Peralta-Ruiz[2,3‡], Donatella Usai[4], Francesca Maggio*[2], Junior Bernardo Molina-Hernandez[2], Davide Rizzo[5], Francesco Bussu[5], Salvatore Rubino[4], Stefania Zanetti[4], Antonello Paparella[2], Clemencia Chaves Lopez*[2]

Table S1. Main volatile compounds identified in *Ruta graveolens* essential oil.

Compound	%	Functional groups	%
2- undecanone	42.6	Ketones	78.7
2-nonanone	23.5	Sesquiterpenes	5.6
2-decanone	4.0	Alcohols	4.2
2-nonanol	3.0	Esters	4.8
2-dodecanone	2.9	Sesquiterpenoids	1.3
Isodecanone	2.6	Furanocoumarins	1.4
2-tridecanone	2.5	Monoterpene ketones	0.3
Benzyl acetate	1.7	Total unidentified components	3.7
1-Methylheptyl acetate	1.3		
2-Undecanol	1.1		
Humulene	1.1		
(-)-Aromadendrene	0.9		
trans-β-Caryophyllene	0.8		
Viridiflorol	0.8		
Nonyl acetate	0.7		
Benzyl 2-hydroxybenzoate	0.5		
σ-cadinene	0.5		
